# The Rural Nursing Workforce Hierarchy of Needs: Decision-Making concerning Future Rural Healthcare Employment

**DOI:** 10.3390/healthcare9091232

**Published:** 2021-09-18

**Authors:** Daniel Terry, Blake Peck, Ed Baker, David Schmitz

**Affiliations:** 1 School of Health, Federation University Australia, Ballarat 3350, Australia; b.peck@federation.edu.au; 2 Center for Health Policy, Boise State University, Boise, ID 83725, USA; ebaker@boisestate.edu; 3 Department of Family and Community Medicine, University of North Dakota, Grand Forks, ND 58202, USA; david.f.schmitz@und.edu

**Keywords:** employment, decision-making, rural, nursing, nursing student, students, workforce, Community Apgar

## Abstract

Addressing nursing shortages in rural areas remains essential, and attracting nursing graduates is one solution. However, understanding what factors are most important or prioritized among nursing students contemplating rural employment remains essential. The study sought to understand nursing student decision-making and what aspects of a rural career need to be satisfied before other factors are then considered. A cross-sectional study over three years at an Australian university was conducted. All nursing students were invited to complete a Nursing Community Apgar Questionnaire to examine their rural practice intentions. Data were analyzed using principal component analysis, and mean scores for each component were calculated and ranked. Overall, six components encompassed a total of 35 items that students felt were important to undertake rural practice after graduating. Clinical related factors were ranked the highest, followed by managerial, practical, fiscal, familial, and geographical factors. Maslow’s Hierarchy of Needs provided a lens to examine nursing student decision-making and guided the development of the Rural Nursing Workforce Hierarchy of Needs model. Each element of the model grouped key factors that students considered to be important in order to undertake rural employment. In culmination, these factors provide a conceptual model of the hierarchy of needs that must be met in order to contemplate a rural career.

## 1. Introduction

Seeking to address health workforce shortages in rural and remote areas has been the principle focus of many governments. This led to greater education and training to occur in rural and regional centers to attract more graduates into rural areas [1,2]. The challenges of sustaining a strong rural health workforce are not new and one that many countries have grappled with and continue to address as they seek to develop more creative ways to resolve this workforce shortfall [2,3,4,5,6,7,8,9]. Such endeavors have yielded insights into the recruitment and retention of health professionals in rural contexts; however, very little research has focused specifically on the factors that drive students and newly graduated health professionals, such as nurses seeking rural employment [10,11].

Although more research is necessary, evidence exists regarding factors that contribute toward health professionals working in rural contexts longer term. For example, students from rural backgrounds, those who have had longer periods or rural clinical placement, and those who had contemplated rural employment post-graduation, remain ideal candidates [7,12,13,14,15]. Therefore, if a nursing student only meets certain elements of these key attributes, it would be reasonable to assume that they would be less likely to undertake a rural career. However, the question that remains is what other factors may influence nursing students who do not come from a rural background, and who have had limited clinical placement experiences in rural areas, to geographically relocate there? As such, is likely that as we understand the factors that students consider important for taking up rural practice, this can support rural recruitment and retention further.

Nursing programs can increase the number of experiences students have in rural areas through the placement elements of their bachelor-level programs, and it is possible to seek opportunities to recruit students who have resided in a rural area. Nonetheless, current research [7,12,13,14,15] suggests that these approaches are only a few of the complex mixture of factors that impact rural employment [7,12,13,14,15]. Additionally, there is a need to gain a greater understanding of the decision-making process among nursing students regarding what factors are important to them when considering entering the health workforce within a rural or remote context. 

Although the first two factors, rural background and rural clinical placements, are well understood, the process of student decision-making, and the interplay among factors that are considered most important to students when considering a rural career remains lacking [16,17]. Several studies have examined ‘decision-making’ factors among health professions [8,9,11,18] with some studies identifying several key factors among these students [14,16,19,20]. Despite these findings, studies remain bereft of consideration as to the interrelatedness and prioritization of these factors among student populations, and more specifically, among nursing students.

Across several health professions, both financial and non-financial incentives are suggested to increase attraction to rural healthcare employment [21,22]. Some examples include meeting the needs of the health professional’s family [23], and the proximity or ease of access to larger, more urbanized centers as factors of attraction [23,24,25]. It is suggested these are all contributing to the decision-making process among many health professionals [8,9,11,26,27], and is reflected by the ways rural and remote health services advertise healthcare employment [28]. 

The work of Prengaman et al. [11,26], followed by Terry et al. [16,17,19], have identified a set of 50 factors, encapsulated within the Nursing Community Apgar Questionnaire (NCAQ), that both registered nurses and nursing students consider important when contemplating working in rural practice. The NCAQ was developed to quantify resources and capabilities of rural communities to recruit and retain healthcare professionals [11,26]. As such, it is used to highlight the relative strengths and challenges of a community’s overall capacity to recruit and retain healthcare staff and thereby supports health facilities to develop achievable long-term employment outcomes [11,18,26]. A modified version of the questionnaire, specifically developed for healthcare students, has been validated to understand nursing student’s intentions to undertake rural practice after graduation [16,17,19].

The challenge is that the initial research of Prengaman et al. [11,26], followed by Terry et al. [16,17,19], has only assumed or surmised where or why nurses and nursing students place certain needs or factors above other aspects of rural life and practice. Their work identifies each individual factor that is considered important by students and nurses, however, does not contextualize these factors or groups of factors within any level of priority. As such, there remains a lack of understanding into what groups of factors are most vital among each of the student cohorts that were examined. In other words, which of the factors are most prioritized by students, and need to be satisfied first before other important factors are even considered, as part of the rural employment decision-making process [7]. 

The benefits of understanding these factors and the priority or ‘hierarchy’ in which they are placed or ranked by student and novice nurses remains crucial for rural health services. Prioritization is key, as rural health services have limited resources; are challenged by a smaller pool of potential employees; and have longer times to fill vacancies when competing with larger, often more attractive, health services [29,30].

### Aim, Purpose, and Research Questions of the Study

The aim of the study was to provide an understanding of the hierarchy of decision-making among nursing students who are currently studying their baccalaureate degree.

As such, the research question centers on what factors are most important or are prioritized for undertaking a rural nursing career that must be satisfied before student and novice nurses contemplate other less important aspects of rural practice. The purpose of the study was to develop a Rural Nursing Workforce Hierarchy of Needs model, conceptualizing those characteristics that must be fulfilled in order for nursing students to contemplate a rural career.

## 2. Materials and Methods

To examine the decision-making hierarchy of importance among Bachelor of Nursing students regarding rural nursing careers, we used a cross-sectional design. The longitudinal study collected individual student data annually over three years at an Australian university with rural, regional, and peri-urban campuses that provided a diversity of perspectives concerning future rural practice. 

### 2.1. Sample

All nursing students studying a three-year bachelor’s degree over the three-year period, from 2018 to 2020, at the university were invited in the mid-semester break to complete an online questionnaire that examined their rural practice intentions (Table 1). It is vital to note that most students may have been asked to participate in the study more than once over the three-year time period. This demonstrates a higher number of participation requests (*n* = 6738) than actual students studying the degree over this time-period (*n* = 4038). For example, second- or third-year students in 2019 and 2020 may have been invited to participate more than once over the two- to three-year period. However, only their most recent responses were included in this study. To achieve this, the linking of questionnaires between multiple years was undertaken by using participant birthdate and postcode. This would ensure most recent responses were analyzed while maintaining participant anonymity. It must be noted that international students were included in the study as in most cases international students, who were on student visas, were aiming towards permanent residency and to work in Australia. Overall, the sample size required (*n* = 363) was calculated to have power to detect a 5% absolute difference within and between groups, alpha (2 tailed) = 0.05, margin of error = ±5%.

### 2.2. Data Collection Tool

Data were collected using a questionnaire that included several standardized demographic questions such as gender, year of birth, place of residence, employment status, income, potential nursing specialization, future work locations, and marital status. The rural background of a student was self-nominated when asked to designate where they grew up. Students were to select, inner city metropolitan, outer suburb metropolitan, large town or regional center, small town, or on a property or farm. This measure of rurality remains a validated tool used for national reporting among healthcare students who undertake clinical placements, and therefore was considered the most appropriate here [14,16]. In addition, the questionnaire included a modified version of the NCAQ, with modifications consisting of slight wording changes for the Australian context. The NCAQ demonstrates good reliability with a Cronbach alpha of 0.96, and good face and content validity [11,16,17,26]. It must be noted that the original NCAQ was administered in the United States as a paper-based questionnaire using a structured interview approach [26], while the initial Australian version was developed, piloted, and validated as an online questionnaire [11].

The NCAQ is composed of 50 individual factors pertaining to nurse practice intentions designed for recruitment and retention in rural areas. For each factor, the tool establishes the advantages or challenges, as well as the level of importance participants place on working in rural areas. The 50 factors are classified into five classes, each containing 10 questions. These five classes comprise geographic factors, economic and resource factors, management and decision-making factors, practice environment and scope of practice factors, and community and practice support factors [11,16,26]. The NCAQ asks participants to rate the level of importance they place on each of the 50 factors with ratings consisting of a four-point scale (very important, important, unimportant, very unimportant). Questions pertaining to the advantages or disadvantages of each of the 50 factors were unrelated, as students were not registered nurses or managers currently in rural practice [16]. The questionnaire tool took between 15 and 25 min to complete.

### 2.3. Data Collection

Data collection occurred mid-year in 2018, 2019, and 2020 in the mid-semester break of each study year. Administration staff supported the study by sending electronic letters on behalf of the researchers to all nursing students via email. This was to maintain anonymity of students and to reduce coercion. The letters included a web link to the information regarding student participation and undertake the survey on-line. Follow-up recruitment emails were sent from administration staff at weeks 1, 2, and 4 from the time of the first invitation. It must be noted that if a student had completed a questionnaire multiple times within the three-year period, the most recent completed questionnaire was included. Moreover, if the NCAQ was incomplete, the student’s data were excluded. Overall, this approach provided a unique number of most recently completed NCAQs scores over the study period.

### 2.4. Data Analysis

Data were cleaned, checked, and analyzed using and Microsoft Excel (Version 15.25.1, Microsoft, Redmond, WA, USA) and Statistical Package for the Social Sciences (SPSS, Version 25.0, IMB, Armonk, NY, USA) [31]. As outlined by the procedure by Prengaman et al. [11], data were scored by assigning quantitative values to the four-point scale according to the participant’s perceived importance (very important = 4, important = 3, unimportant = 2, very unimportant = 1). These scores of importance for each factor were then divided by the number of participants to produce an overall mean score as described elsewhere [11,16,17,20].

In addition to the NCAQ scoring, the 50 items were analyzed using principal components analysis (PCA) using Varimax rotation to extract the maximum amount of variance across the NCAQ factors [32]. Data suitability was examined before PCA was conducted, whereby items were excluded if loading of coefficients was less than 0.50 [32]. In addition, the Kaiser–Meyer–Oklin measure of sampling adequacy was shown to be at 0.974, above the recommended value of 0.6, and Bartlett’s test of sphericity was 0.000 and supported the factorability of the correlation matrix [32]. The mean scores for each component were calculated, and components were ranked highest to lowest to provide insight into the individual components relevant to how important students felt each factor was in terms of taking up rural employment. 

### 2.5. Theoretical Frameowork to Understand the Data

To help guide our understanding, Maslow’s [33] Hierarchy of Needs was utilized as a framework or lens to examine the decision-making of nursing students considering rural practice. Maslow’s Hierarchy of Needs has been used across various disciplines and settings to understand human behavior and decision-making [34]. Examples include education and academic success [34], business and motivating employees [35], patient care [36], information technology [37], and areas such as food and tourism [38].

Maslow’s Hierarchy of Needs hypothesizes that needs are separated into five levels of priority and demonstrates these various groups of needs visually as a triangle, with the most basic lower-level needs at the base, with higher level needs ascending the triangle [36,39]. For example, basic physiological needs for human life and survival include food, water, and shelter, while the next group of needs or goals, once the physiological needs are met, include health, employment, family, and social stability [33,39]. Once the prioritized needs are satisfied, other needs become next-order priorities, such as love and belonging, and so on. Maslow indicates that unless the most basic needs are satisfied, higher-level needs are less thought about as the focus of individuals or communities [33,34,35].

### 2.6. Ethical Considerations

Approval for the study was granted by the Federation University Australia Human Research Ethics Committee (approval #18-017). The invitation to participate in the anonymous survey was sent in the mid-year break to reduce any risk of bias or coercion while undertaking their studies. Students consented to their participation by freely completing the questionnaire. Students did not receive any incentives for participation.

## 3. Results

The online questionnaire was sent via email to a total of *n* = 4038 individuals who were first-, second-, or third-year students undertaking a Bachelor of Nursing degree in 2018, 2019, and 2020. Among the nursing students invited to participate, *n* = 885 (21.91%) responses for the NCAQ were received, well above the sample size required (*n* = 363). After excluding for incomplete and any multiple NCAQ completions among students (*n* = 81), there were *n* = 804 unique NCAQ results from students across the three years. Across the three years, student demographics were similar; however, it was noted that a greater proportion of international students participated in the 2020 survey (37.46%) when compared to previous years. In addition, a greater proportion of students who grew up in an inner-city metropolitan area also participated in 2020 (11.46%) compared to student participants from previous years (Table 2).

**Figure 1:**
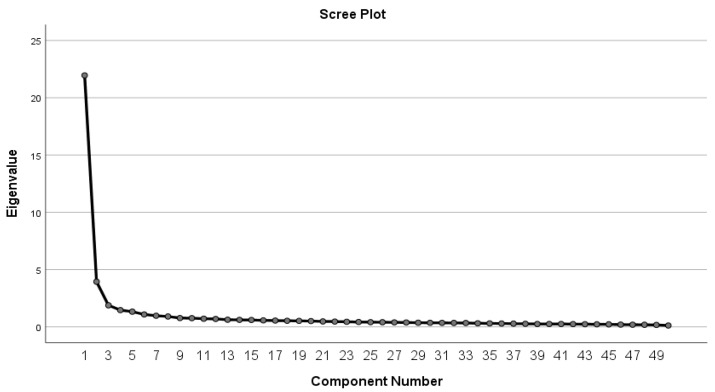
Principal component analysis scree plot.

The 50 items from the NCAQ were subjected to PCA with a Varimax rotation, as previously described. To identify and label each component, we undertook an additional exploration of the highest loaded items. The PCA revealed six components that were shown to have eigenvalues above 1, as shown in Figure 1 and Table 3.

The analysis demonstrated that the six components encompassed a total of 35 NCAQ items that were retained due to their higher levels of predictability to nursing students’ level of importance placed on undertaking rural practice after graduating. These six components explained 63.28% of the total variance of indicators of NCAQ. The six components were identified and labelled as clinical, fiscal, practical, geographical, managerial, and familial, as outlined in Table 4.

After the PCA was completed, the mean scores for each component were calculated and ranked highest to lowest to indicate how important students felt each component was to take up rural employment. As such, it was indicated that clinical related factors were ranked the highest, followed by managerial, practical, fiscal, familial, and geographical factors, as outlined in Table 5 and discussed in detail below.

### 3.1. Clinical

The clinical component, identified as the most important element of considering rural practice, included 13 NCAQ factors which centered on many clinical aspects of rural employment. These emphasize how important the practice environment may be for novice nurses. As such, there is an emphasis on the provision of quality, ethical care, and evidence-based care. In addition, other elements of importance include workforce morale and stability, partnerships between nurses and other medical staff, and how well the workplace and community as a whole is accepting of new nurses to the health service.

### 3.2. Managerial

In line with the clinical aspects of the decision-making process, nursing students considering rural practice employment also contemplate several managerial aspects, such as being included and involved in decision-making process of the health service in terms of equipment, technologies, and processes. Novice nurses want to be a part of the solution to the challenges they may see or encounter in their everyday practice as they provide care, which is further emphasized when nursing students can see opportunities or pathways for professional development and advancement. Lastly, the opportunity to contribute to the health service through teaching and mentoring is highlighted as also being vitally important.

### 3.3. Practical

After considering the clinical and managerial aspects in the decision-making process and being professionally fulfilled, several practical elements of the nursing role and health service then become important to nursing students. In this sense, what now becomes important is how the health service and employment environment may be perceived by those inside or outside of the health service or community. This perception of the health service and community is also related to nursing students seeking employment where they feel they are welcomed, needed, supported, and appreciated by their colleagues, but also by the wider community. Part of the decision-making is about the practical opportunities for growth and expansion within their career. Students find it important when considering rural practice in that there are career pathways beyond the ward where they may first commence. Opportunities to access further education, as well as specialize and expand their careers within the rural context, is vital. Although it may be considered less important than some of the more immediate clinical and managerial aspects of rural employment, they are certainly considered here as part of the decision-making process.

### 3.4. Fiscal

Considered important when contemplating any employment opportunity, the fiscal aspects of the decision-making process were shown to be less important. However, a key consideration was the availability of a moving allowance for relocating. This may be vital, given a student, new to nursing, may not have adequate resources prior to commencing, and this therefore may directly impact on their capacity to re-locate. Moving allowance may not always be financial payment, and it can also include being supported in the move itself or related to short-term accommodation while a more permanent solution is enabled. Along with this aspect of the decision-making process concerning moving is the aspect of housing, where cost of living, availably, or affordability are essential, wherein high demand or costs impacts disposable income of the individual. Lastly, there are key aspects beyond the associated costs of moving and living, which are related with the salary itself. Benefits beyond salary that may be considered essential include study days or educational bursaries, as well as opportunities to undertake different shifts where higher salary may be earned.

### 3.5. Familial

Once the financial aspects of the decision-making process are addressed, it is noted that familial elements become important for students. In this sense, there were three key factors determined to be most important from the student’s perspective. These were adequacy and access to schools for children; opportunities for employment and satisfaction of a spouse or partner in the geographical location; and, for the nurse themselves, access to readily available childcare centers, which may be close to the health service and meet the needs of healthcare workers.

### 3.6. Geographical

Lastly, students highlighted that the geographical component may be important in their decision-making; however, being much less important than other components previously highlighted. Nevertheless, the diversity of patients and health conditions may be observed as important to maintain a diversity of skills or practices. In addition, other factors including the size of the community and the ability for socializing in the community outside of the workplace is considered vital. In some respects, opportunities and easy access to recreational activities often associated with many rural communities is also considered important. Finally, access to larger communities which are an ‘easy’ commute from the rural area and health service also contributes to the decision-making process. As such, when potential employees are recruited from an urban location, it is suggested that access to an urban location lowers the anticipatory stress when considering re-location to a rural area, wherein the access to metropolitan areas is considered ‘not so far away or different’ in this sub-population of potential recruits. Although considered important, geography remains much less important as a priority when compared to other components already identified.

## 4. Discussion

Overall, the study sought to understand nursing student decision-making and what aspects of a rural career need to be satisfied before other factors are then considered. An online questionnaire was sent via email to a total of *n* = 4038 individuals and *n* = 804 unique NCAQ were completed among students across the three-year period. Data were analyzed using principal components analysis, and mean scores for each component were calculated and ranked. Overall, six components encompassed a total of 35 items of importance that students placed on undertaking rural practice after graduating. Clinical related factors were identified as the most important element of considering rural practice, followed by managerial, practical, fiscal, familial, and geographical groups of factor elements.

Maslow’s [33] Hierarchy of Needs was applied as a framework or lens to guide the understanding of the key elements and their levels of importance among nursing students considering rural practice. Within the healthcare environment, the work undertaken by Benson and Dundis [35] further provides insights into how Maslow’s hierarchy assists in guiding the understanding of what was occurring among healthcare employees. For example, when adequate wages or more central fundamental needs were satisfied, only then were feelings of safety, security, feeling needed, opportunities for self-development, and growth then noted as motivating and associated with a heightened commitment to their employer [35].

Although not seeking to replicate what has been achieved by Maslow [39], Benson and Dundis [35] and others [34,36] purposed the use of the tenets of Maslow’s work to provide a framework to better understand nursing student decision-making. In this sense, the framework helps to conceptualize those factors students prioritize as most important when compared to other factors that are felt to be less important when considering rural practice. For example, if factor A is more important that factor B, then among potential employees, factor A has a higher priority above factor B and a hierarchy of the factors exists in terms their importance among students. 

**Figure 2:**
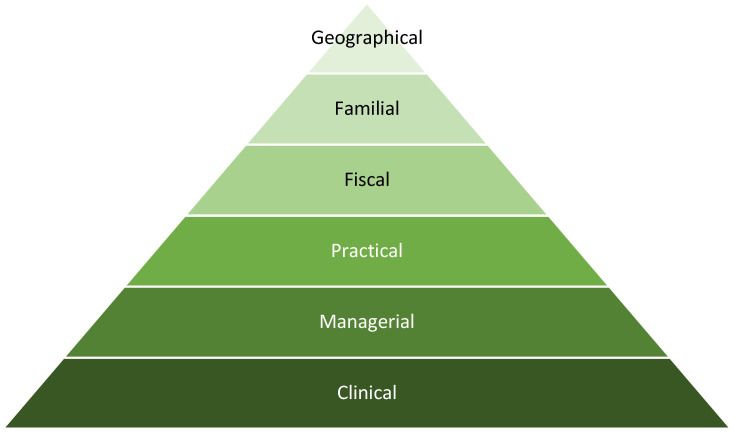
The Rural Nursing Workforce Hierarchy of Needs model or hierarchy of workforce need.

These findings have led to the development of the Rural Nursing Workforce Hierarchy of Needs (Figure 2). It is where each element of the model, namely, clinical, managerial, practical, fiscal, familial, and geographical are made up of groups of key factors that students consider and prioritize to be important in undertaking rural employment. Each element, made up of their respective factors, have differing levels of importance when compared to each other, and within this hierarchy, are prioritized as needing to be addressed before other factors are then more fully considered. Although all factors may be viewed simultaneously, it is not until the lower level and first-order elements are met that other higher-level factors will be fully considered in the decision-making process.

It must be noted that not all six elements and corresponding factors must be addressed or satisfied for an individual to consider rural employment. Some individuals may only require clinical and managerial elements before they are satisfied to make a decision, while others, depending on life circumstances, such as marital status, age of children, and relative income needs, may consider the clinical through to familial elements as part of their decision-making. However, what remains constant is the hierarchy of the elements in the decision-making process. If the more fundamental or first-order elements, such as clinical aspects of the workplace, as outlined in Table 3, are not addressed, or remain inadequate, then higher-level elements, such as fiscal, familial, or geographical aspects, remain extraneous.

In this sense, the Rural Nursing Workforce Hierarchy of Needs model seeks to provide an understanding of what nursing students consider to be fundamental needs in their contemplation of rural employment. Further, the model seeks to better inform health services in where to focus their energies and limited resources to meet these needs, before focusing on higher-level needs, which nursing students will not consider until their basic needs are met. As such, the hierarchy of the Rural Nursing Workforce Hierarchy of Needs model indicates the level of importance or necessity of fulfilling the more basic first-order needs before higher level needs, as has been used elsewhere in healthcare decision-making [36]. As such, from a health service’s perspective, it would therefore remain fruitless to focus energy, time, and recourses among health services on ‘selling’ geographical factors if nursing student’s fundamental needs within the Rural Nursing Workforce Hierarchy of Needs model, such as clinical factors, remain unsatisfied. 

A major finding of this study is that in order for nursing students to consider a career in a rural healthcare setting, the clinical needs must be met. For example, a workplace that emphasizes patient safety and high-quality care, positive relationships, communication among nurse generations, workplace culture, a supportive working environment, and autonomy and respect, as outlined in Table 3, remain the most import factors among students. Specifically, this means that an agency must be focused on providing high-quality healthcare while at the same time establishing and maintaining a strong sense of morale amongst staff. These same factors have also been demonstrated to be vital with nurse retention and workplace satisfaction [24,40,41]. Nursing students engage with a myriad of different clinical venues, clinical staff, and workplace cultures throughout their undergraduate program. According to Kramer et al. [42], nursing students attend professional-practice experiences with high expectations of what to anticipate when entering as graduates. Students anticipate that the work environment will readily support the establishment of working relationships needed to deliver quality patient care, as experienced during the academic and practical placement learning [42]. On the basis of their experiences, students are well placed to discern underlying incivility and will readily make decisions about their future employment opportunities on the basis of what will ‘fit for them’.

With less brand, employer, or service loyalty than perhaps what once existed within nursing, the new generation of nurse recognizes that they are in demand and are actively mobile in their search for the best or a better work environment [43]. Research has consistently shown that focusing on efforts to improve satisfaction with the work environment leads to effective retention of early career nurses [44]. As with Maslow’s hierarchy, so too, the Rural Nursing Workforce Hierarchy of Needs model would suggest that if the clinical factors for a student are not being met by an agency, then all subsequent factors are irrelevant [33]. Nursing students first and foremost want to know those aspects of a workplace, embodied here as the clinical aspects of rural employment, are in place and going to work for them.

If the clinical aspect of rural employment is adequate or meets the needs of the student, then the next priority is the managerial factors. Having positive relationships with management staff may directly impact on one’s workplace satisfaction, something that has been extensively explored in the literature [17,30,45]. However, the characteristics of managerialism being explored here are in relation to the student nurse perceiving that they are a valued contributor to the decision-making process as well as future opportunities for leadership. Previous research supports this finding amongst both student and early career nurses, identifying that where the management style of the ward was open and inclusive in decision-making satisfaction was improved [43]. Students want to feel empowered within the clinical setting, and being involved in the decisions that directly affect the everyday activity enhance that sense of belonging and meet the need of managerial factors [45]. Again, where there is little room for professional growth in the workplace, then they are less likely to choose or even stay regardless of practical, fiscal, and family benefit [46].

What we termed practical factors in this study closely follow the managerial aspects of the workplace Rural Nursing Workforce Hierarchy of Needs model. As such, if the practical aspects of rural employment are not satisfied, such as the perception of the community and the health service, and the nurse being welcomed, needed, supported, and given opportunities for growth, then factors such as fiscal, familial, and geographical will continue to be immaterial and remain frivolous to promote. The need for recognition and a sense of belonging to the broader community is an indicator of ongoing rural employment that has also been shown to come with its own potential issues. While students might wish to be welcomed and to belong to a community, Jones et al. [47] found that early-career nurses were struck by the blurring of personal and professional boundaries that came from working in smaller rural areas. We suggest that upwardly mobile student nurses who may or may not have or even contemplated home ownership or families are seeking what ‘fits for me’, and if clinical, managerial, and practical aspects of the workplace do not fit, then any other factors remain unwarranted or trivial.

Although these findings are founded on students in their first, second, or third year of undertaking their baccalaureate degree program, it has been indicated by Terry et al. [48] that NCAQ scores among students did not significantly differ to their scores 18–24 months after graduating. This suggests that what is considered important among students will also be reflective what novice nurses consider important within the first two years of workforce experience. Further, it must be noted that there were a number of different cohorts within the study sample in terms of personal, social, economic circumstances, and life stages. It may be suggested that those who are more mature participants may be well established with financial responsibilities and family commitments, and therefore may preclude their practical capacity to contemplate more rural employment [8,17,48]. However, the Rural Nursing Workforce Hierarchy of Needs model would propose that regardless of these heterogenic circumstances, clinical, managerial, and practical factors remain essential to be met above other key factors yet require further investigation.

Regardless, the Rural Nursing Workforce Hierarchy of Needs model may provide an additional layer of understanding regarding the retention of novice nurses, beyond rural contexts. It has been demonstrated that novice nurses leave their employment due to issues of disempowerment, poor working conditions, and difficult work relationships and management, in search for other nursing employment. They search for positions where there is a ‘better fit’, which is in line with what novices are seeking within Rural Nursing Workforce Hierarchy of Needs model [27,49].

Further, when employed nursing staff were examined in the Prengaman et al. [11] NCAQ study, and students in this study, individual responses pertaining to geographical and economic factors within the NCAQ were the least important and were similar between the two cohorts. However, it is noted that differences exist between cohorts regarding community and practice support responses, wherein these differences may be due to the age variations or the dissimilar life stages of the two varying groups [8,9]. Despite these differences between cohorts, the findings highlight than both management and practice support responses were rated by both nursing staff and students as highly important. This suggests the NCAQ questions pertaining to the managerial and clinical elements are at the forefront of both seasoned nursing staff and nursing students, where little distinction between the two groups may exist [41]. However, in the study conducted in Laos, a number of differences between nursing students compared to practicing nurses regarding what they considered important to take up rural employment was evident [50]. As such, the Rural Nursing Workforce Hierarchy of Needs model and its applicability to the recruitment of more experienced nurses requires further testing and investigation.

### Limitations

Research has routinely demonstrated that those student nurses who have grown up in a rural and remote are more likely to consider and engage with a career in a more rural area once qualified. The students invited to participate in this study were from a myriad of rural, regional, and peri-urban areas that may have implications for generalizing the findings, as many universities are located in more metropolitan or urban city centers. All students, except three (*n* = 3), within the cohort examined were from rural, regional, and peri-urban background and/or had experienced one of more of their clinical placements within a rural or regional context. Although it has been shown rural background and placement experience influences a student’s perception and intention to work in a rural area, respondents may have rated their importance of factors on the basis of assumptions about those characteristics of rural practice. It must be noted that student respondents may not be representative of the entire cohort given the lower response rate (14.6–18.5%). Despite this, the methodology adopted for the analysis of this data is not affected by lower response rates. Future studies should involve a larger cohort from multiple university locations that represent both urban and more regional sites. Lastly, while the study has created a model of the hierarchy of factors important for nursing student in choosing rural careers, the current study did not ask the nuanced questions of why some factors were more important than other factors. As such, future qualitative research is warranted to explore the needs or factors important for nursing students in choosing rural careers. Future quantitative research is also needed to ascertain if what has been uncovered reflects the broader trends across the healthcare workforce.

## 5. Conclusions

Globally, nursing workforce shortages in rural and remote areas remains a considerable public health issue. As such, the aim of the study was to provide an understanding of the hierarchy of decision-making among nursing students who are currently studying their baccalaureate degree. Within this context, this study continues the development our understanding of those factors that contribute towards health professionals working in rural areas. One of the strongest attributes to correlate with long-term employment in a rural area is having grown up in a rural area. There is, however, a large body of students who do not meet this or other attributes and yet could make a worthwhile population to focus our attention in seeking to attract them to rural practice. The Nursing Community Apgar contributes to the knowledge of what both current nurses and student nurses consider important when contemplating a nursing career in rural settings. However, we did not have any sense of how these factors are contextualized or grouped together in a hierarchy in terms of what factors take priority over others among nursing students. This study has identified the Rural Nursing Workforce Hierarchy of Needs model for conceptualizing those characteristics or needs that must be met in order for a nursing student to then contemplate a rural career. 

It has been identified that there is a need for health services to look beyond salary, access to larger or urbanized centers, and the natural beauty of the landscape and focus their efforts on meeting what students and even novice nurses are seeking. This includes a supportive practice environment that is stimulating and fosters empowerment and growth as an employee. Further, a healthcare service that provide high quality care in a setting that has a high degree of workforce morale and is welcoming to new staff is a ‘non-negotiable’ in the recruitment stakes. Therefore, employment must be better advertised or promoted among students and novice nurses in a way that demonstrates the benefit of being part of the health services—a place to find meaning and purpose within the rural context. Overall, the employment of the new and rising workforce is about the ‘fit for the individual’, while also addressing the ‘fit for the health service’. As such, when working in harmony, there is a mutual and reciprocal relationship between the employee and employer, where there is a meeting somewhere in the middle and the notion of ‘fit for us’ occurs [17].

The modern nursing student recruit is much less interested in perhaps the more stereotypical rewards of rural work such as enticing wages and an idyllic lifestyle, and instead is seeking the best ‘fit for them’ as a member of supportive workforce that values their input and developing expertise. Studies such as this provide healthcare facilities an insight into the hierarchy of those things that must be in place to successfully attract and maintain a nursing workforce among student nurses specifically, while supporting our understanding of more those currently part of the nursing workforce.

In moving forward, it is recommended when seeking to recruit early career nurses, rural health services may benefit from examining their strengths concerning clinical, managerial, and even practical aspects of the service and marketing these. Alternatively, the hierarchy will provide health agencies an opportunity to identify areas of deficit in their own service that can then be addressed to enhance their attractiveness to early career nurses. In addition, services may seek to augment and refocus their approach and energies that are currently focused on examining and advertising geographical, familial, and fiscal factors as an alternative method to improve recruitment and retention endeavors.

More specifically, if seeking to maximize the effective use of scarce resources for recruitment and retention, health services may initially focus on developing clinical factors or advertising these qualities that early carer nurses consider most important. For example, the focus may be on assessing, developing, and advertising clinical factors, such as patient safety and high-quality care; positive relationships and communication between nurses; and a positive and supportive workplace culture that fosters mentoring, staff morale, job satisfaction, and autonomy and respect. Overall, by exploring the most important factors and how they may be applied within the health service will be better in terms of enabling services to invest in those unique factors that improve recruitment and retention.

## Figures and Tables

**Table 1:** Total enrolments over three-year period.

Student Enrolments	2018	2019	2020
First year enrolments	862	921	978
Second year enrolments	788	931	953
Third year enrolments	388	501	416
Total enrolments (Actual)	2038	2353	2347

**Table 2:** Participant demographics.

Demographic Information	Frequency						
	2018		2019		2020		Total
	*n*	(%)	*n*	(%)	*n*	(%)	*n*
Year of program (*n* = 804)							
- First year	61	29.61%	94	34.18%	134	41.49%	289
- Second year	71	34.47%	104	37.82%	102	31.58%	277
- Third year	70	33.98%	77	28.00%	45	13.93%	192
- Missing	4	1.94%	0	0.00%	42	13.00%	46
Gender (*n* = 804)							
- Female	181	87.86%	199	72.36%	248	76.78%	628
- Male	21	10.19%	22	8.00%	31	9.60%	74
- Other	0	0.00%	2	0.73%	2	0.62%	4
- Missing	4	1.94%	52	18.91%	42	13.00%	98
Age (years) (*n* = 804)							
- Under 20	24	11.65%	14	5.09%	57	17.65%	95
- 20–30 years	55	26.70%	84	30.55%	88	27.24%	227
- 30–39 years	59	28.64%	78	28.36%	95	29.41%	232
- 40–49 years	43	20.87%	46	16.73%	64	19.81%	153
- 50 years and over	9	4.37%	23	8.36%	19	5.88%	51
- Missing	16	7.77%	30	10.91%	0	0.00%	46
Born in Australia (*n* = 804)							
- Yes	163	79.13%	164	59.64%	160	49.54%	487
- No	39	18.93%	59	21.45%	121	37.46%	219
- Missing	4	1.94%	52	18.91%	42	13.00%	98
Marital status (*n* = 878)							
- Single	72	34.95%	73	26.55%	104	32.20%	249
- Married/partnered	112	54.37%	127	46.18%	150	46.44%	389
- Divorced/separated	11	5.34%	12	4.36%	17	5.26%	40
- Other	6	2.91%	2	0.73%	10	3.10%	18
- Missing	5	2.43%	61	22.18%	42	13.00%	108
Highest level of education (*n* = 804)							
- Secondary school (year 12 or less)	29	14.08%	66	24.00%	112	39.86%	207
- Vocational or trade training	141	68.45%	123	44.73%	126	44.84%	390
- Bachelor’s degree or above	34	16.50%	25	9.09%	38	13.52%	97
- Other	2	0.97%	4	1.45%	5	1.78%	11
- Missing	0	0.00%	57	20.73%	42	13.00%	99
Employment status (*n* = 804)					281		
- Not in paid labor force	36	17.48%	29	10.55%	20	6.19%	85
- Casual employee	57	27.67%	88	32.00%	66	20.43%	211
- Part-time employee (>38 h week)	80	38.83%	103	37.45%	126	39.01%	309
- Full-time employee (38 h a week)	22	10.68%	38	13.82%	29	8.98%	89
- Missing	11	5.34%	17	6.18%	82	25.39%	110
Current after-tax income (AUD) a week (*n* = 804)							
- Less than $400	85	41.26%	94	34.18%	70	21.67%	249
- $400–$799	65	31.55%	110	40.00%	114	35.29%	289
- $800–$1499	26	12.62%	40	14.55%	45	13.93%	111
- $1500–$3000	3	1.46%	5	1.82%	8	2.48%	16
- Do not want to answer	23	11.17%	0	0.00%	27	8.36%	50
- Missing	4	1.94%	26	9.45%	59	18.27%	89
Where participant grew up (*n* = 804)							
- Inner city metropolitan	10	4.95%	17	6.18%	37	11.46%	64
- Outer suburb metropolitan	48	23.76%	47	17.09%	64	19.81%	159
- Large regional center	45	22.28%	50	18.18%	47	14.55%	142
- Small town	60	29.70%	79	28.73%	86	26.63%	225
- On a property or farm	30	14.85%	23	8.36%	30	9.29%	83
- Other	9	4.46%	7	2.55%	17	5.26%	33
- Missing	4	1.94%	69	25.09%	79	24.46%	162

**Table 3:** Principal component analysis.

Component	Initial Eigenvalues			Extraction Sums of Squared Loadings			Rotation Sums of Squared Loadings		
	Total	% of Variance	Cumulative %	Total	% of Variance	Cumulative %	Total	% of Variance	Cumulative %
1	21.952	43.904	43.904	21.952	43.904	43.904	11.897	23.794	23.794
2	3.943	7.885	51.789	3.943	7.885	51.789	4.998	9.995	33.789
3	1.878	3.756	55.546	1.878	3.756	55.546	4.595	9.191	42.980
4	1.461	2.921	58.467	1.461	2.921	58.467	4.573	9.146	52.126
5	1.324	2.648	61.115	1.324	2.648	61.115	3.340	6.681	58.807
6	1.082	2.164	63.279	1.082	2.164	63.279	2.236	4.473	63.279

**Table 4:** Principal component analysis results for NCAQ.

Factor	Clinical 1	Fiscal 2	Practical 3	Geographical 4	Managerial 5	Familial 6
Emphasis on patient safety/high-quality care	0.834					
Positive relationships/communication among nurse generations	0.800					
Positive workplace culture/supportive working environment that fosters mentoring	0.790					
Job satisfaction morale level	0.754					
Autonomy/respect	0.753					
Manageable workload/increased time with patients	0.720					
Effective partnership between medical and nursing staff	0.702					
Nurse empowerment	0.635					
Ethical climate	0.635					
Evidence-based practice	0.634					
Thorough orientation/preceptorship	0.629					
Acceptance of nurses new to area	0.626					
Nursing workforce adequacy and stability	0.626					
Moving allowance		0.754				
Benefits		0.748				
Salary		0.664				
Shift differential		0.624				
Cost of living		0.620				
Housing availability/affordability		0.552				
Image of rural health care and positive image of job environment			0.679			
Community health/nursing services			0.624			
Sense of reciprocity between nurses and community			0.620			
Welcome and recruitment program			0.609			
Distance education access			0.600			
Demographics/patient mix				0.761		
Social networking				0.754		
Recreational opportunities				0.688		
Access to larger community				0.687		
Size of community				0.639		
Nurses involved in selecting/implementing new technology/equipment					0.606	
Professional development opportunities/career ladders					0.568	
Teaching/mentoring opportunities, involvement/challenge of multiple roles					0.552	
Schools						0.788
Spousal/partner satisfaction						0.624
Day care						0.557
Percentage (%) of variance explained	43.904	7.885	3.756	2.921	2.648	2.164

**Table 5:** Mean score of each nursing student component.

Component	Number of Items	Mean Score
Clinical	13	3.675
Managerial	3	3.483
Practical	5	3.421
Fiscal	6	3.319
Familial	3	3.082
Geographical	5	2.954

## Data Availability

The data presented in this study are available on request from the corresponding author.
